# Relationship between cognition and treatment adherence to disease-modifying therapy in multiple sclerosis: a prospective, cross-sectional study

**DOI:** 10.1038/s41598-022-16790-3

**Published:** 2022-07-20

**Authors:** N. Giedraitiene, V. Taluntiene, G. Kaubrys

**Affiliations:** Clinic of Neurology and Neurosurgery, Institute of Clinical Medicine, Faculty of Medicine, Vilnius University, Vilnius, Lithuania

**Keywords:** Neurology, Multiple sclerosis

## Abstract

Less than half of patients with chronic diseases, including multiple sclerosis (MS), adhere to their prescribed medications. Treatment selection is essential for patient adherence. The aim of this study was to explore the potential factors influencing nonadherence to disease-modifying therapies (DMTs) in MS. This prospective, cross-sectional study was performed at the Multiple Sclerosis Center between 2018 and 2021. In total, 85 patients were eligible for final analysis. Forty-one patient (48.2%) with MS were non-adherent to DMT. Male sex, oral administration of drugs, and longer treatment duration were associated with nonadherence. The mean Expanded Disability Status Scale score did not differ between the adherent and non-adherent patients (p > 0.05). Patients with a higher score on the Symbol Digit Modalities Test, who were receiving self-injection therapy, had shorter treatment duration, and higher disability, were more likely to be adherent to DMT than those without. To minimize nonadherence in patients with MS, the patient’s information processing speed should be considered before DMT initiation, and appropriate treatment options should be discussed.

## Introduction

Treatment options for multiple sclerosis (MS) have greatly expanded recently^1,2^. Improving disease stability and quality of life in patients with chronic diseases requires prolonged and often lifelong medication. Less than half of patients with chronic diseases and MS adhere to their prescribed medications, which precludes the full benefit of treatment, worsens disease outcomes, and accelerates disease progression^3–5^. MS is one of the diseases that, despite the coming of new and short treatment options^1,2^, still requires frequent parenteral or oral administration of disease-modifying therapy (DMT) daily or a few times a week for an undefined extended period.

Poor adherence to treatment in MS reduces the clinical effectiveness of therapy, which can adversely impact disease progression, MS-related hospitalization, and quality of life^6,7^. Hence, treatment selection is essential for patient adherence. Some factors should be considered when making a treatment decision for patients with MS–not only efficacy and safety issues should be considered, but also the route of administration, dosing frequency, patient lifestyle factors, and willingness should be evaluated before DMT administration^8^.

Although there is a need to improve the adherence rate in patients with MS, it is equally important to investigate the relationship between adherence and prognostic factors. Nonadherence to DMT is believed to be caused by numerous factors^9–11^, including perceived lack of efficacy, adverse drug effects, and simply forgetting to inject oneself^3,9–11^. Cognitive impairment has also been associated with nonadherence^12,13^. At least 70% of patients with MS experience mild-to-severe cognitive impairment, most commonly in information processing speed, executive functioning, and visual and verbal memory^14–16^. Despite the cognitive impairment and nonadherence evidence^11–13^, the literature on MS adherence rates lacks recommendations regarding cognitive assessment.

Some studies have shown the relationship between cognitive impairment and nonadherence to DMT for MS^12,13^; however, all of them have failed to examine which cognitive assessment should be performed and how to select potential non-adherent patients based on cognitive assessment before DMT administration. Identifying patient groups that are more likely to be adherent and determining the explanatory factors are essential to designing targeted management strategies, as poor adherence is associated with increased risk of morbidity and mortality^3,4,7^. This study aimed to explore the potential factors influencing nonadherence to DMTs for MS.

## Results

### Patient characteristics

**Figure 1:**
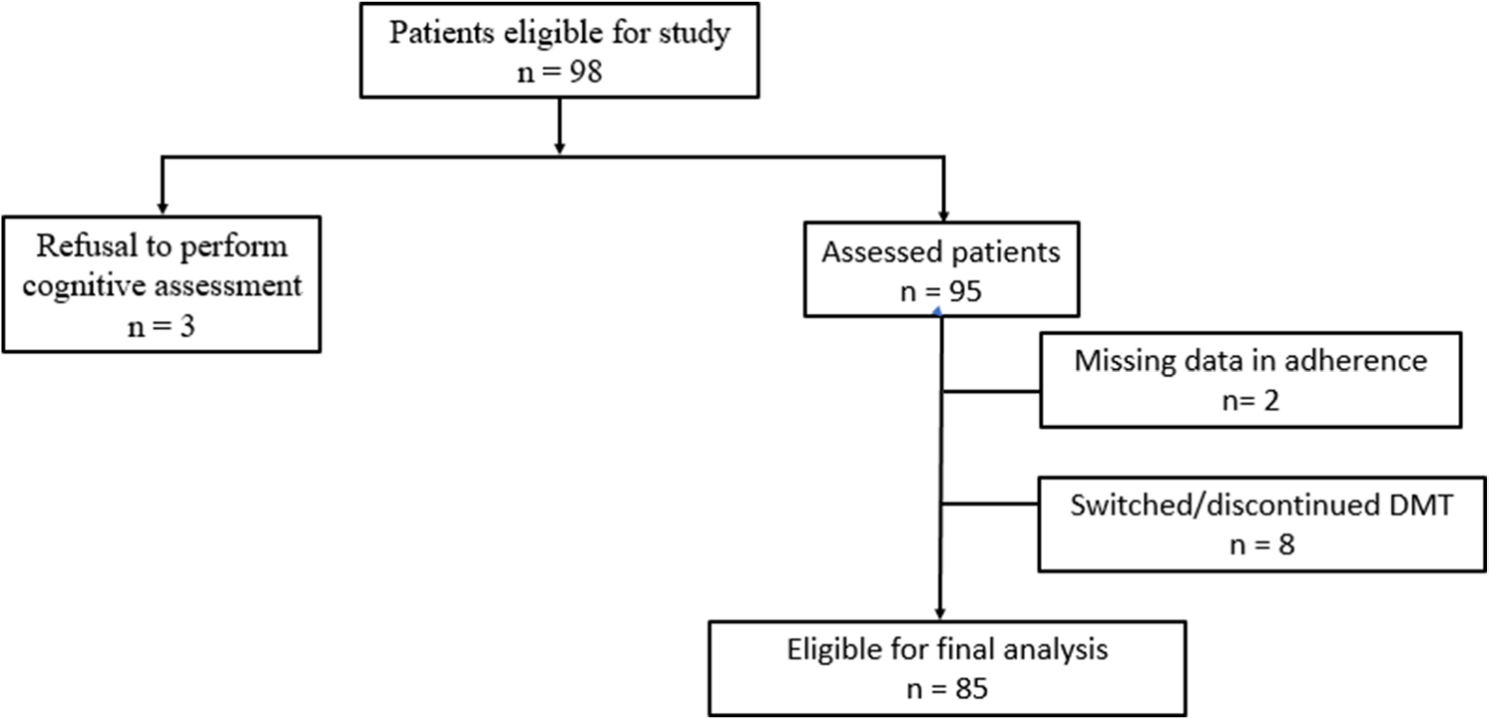
Flowchart of patient selection. DMT, disease-modifying therapy.

Ninety-eight patients were included in this study. Data regarding adherence to DMT and cognitive assessment were available for 85 patients (Fig. 1). Patients’ demographic and clinical characteristics are shown in Table 1.

**Table 1:** Clinical and demographic characteristics of the study participants.

Demographic and clinical variable	N	%
**Sex**		
Female	58	68.2
Age (years)	41.8 ± 10.7	–
Disease duration (years)	12.0 ± 11.8	–
EDSS score	3.4 ± 1.2	–
**Education (years)**		
None/elementary	18	21.2
Secondary	13	15.3
Vocational or higher	54	63.5
**Marital status**		
Married/living with a partner	55	64.7
Single	17	20
Separated/divorced	9	10.6
Widowed	4	4.7
**Professional activity**		
Full-time worker	38	44.7
Part-time worker	14	16.5
Unemployed	33	38.8
**DMT**		
Self-injectable therapy	54	63.5
Oral therapy	31	36.5
**Treatment duration**		
< 12 months	26	30.6
12–24 months	22	25.9
> 24 months	37	43.5
**Comorbidity**		
No	18	21.2
Yes	67	78.8

DMT, disease modifying therapy, EDSS, Expanded Disability Status Scale.

Among the injectable DMTs (n = 54), 22.2% of patients were prescribed glatiramer acetate, 14.8% interferon (IFN)-β-1a intramuscularly, 46.3% IFNβ-1a subcutaneously, 13.0% IFNβ-1b, and 3.7% pegylated form of IFNβ-1a. Among the oral DMTs (n = 31), dimethyl fumarate was prescribed in 41.9% of patients, fingolimod in 38.7%, and teriflunomide in 19.4%.

### Relationship between demographic, disease characteristics, and adherence to DMT

According to the study data of 85 patients with MS who were taking an injectable or oral DMT, the proportion of days covered (PDC) was < 80 in 41 (48.2%) patients. The adherence range was the same in patients up to 45 and older than 45 years of age (p > 0.05). The nonadherence rate was significantly higher in men than in women (p < 0.05). No differences in adherence rates were detected according to disease duration, education level, and professional activity (all, p > 0.05). Oral administration showed a greater lack of adherence, also longer treatment duration (> 12 months) p < 0.05). Non-adherent patients were associated with an increased frequency of relapse in the post-index 12-month period (p < 0.05) (Table 2).

**Table 2:** Adherence rate in patients with MS according age, sex, education level, professional activity, and form of drug administration.

	Adherent patients	Non-adherent patients	p-value
**Age**			
< 45 years	24	22	χ2 = 0.007 p > 0.05
≥ 45 years	20	19	
**Sex**			
Male	10	17	**χ2 = 3.44 p < 0.05**
Female	34	24	
**Education level**			
None/elementary	8	10	χ2 = 1.996 p > 0.05
Secondary	5	8	
Vocational or higher	31	23	
**Professional activity**			
Full-time worker	20	18	χ2 = 1.902 p > 0.05
Part-time worker	5	9	
Unemployed	14	19	
**DMT**			
Self-injectable therapy	32	22	**χ2 = 3.331 p < 0.05**
Oral therapy	12	19	
**Treatment duration**			
< 12 months	18	8	**χ2 = 4.576 p < 0.05**
≥ 12 months	26	33	
**Relapse**			
Relapse in the last 12 months	18	25	**χ2 = 3.419 p < 0.05**
No relapse in the last 12 months	26	16	

Significant values are in [bold].

EDSS, Expanded disability status scale; DMT, disease-modifying therapy; MS, multiple sclerosis.

### Neurological disability, cognitive impairment, and adherence to DMT

The mean Expanded Disability Status Scale (EDSS) score did not differ between the adherent and non-adherent patients (p > 0.05). The scores of information processing speed and visuospatial memory were significantly lower in non-adherent patients than in adherent patients (p < 0.05), whereas the scores for verbal learning did not differ between the groups (p > 0.05) (Table 3).

**Table 3:** Cognitive scores and neurological disability in adherent or non-adherent patients with MS.

	Adherent patients	Non-adherent patients	p-value*
EDSS score	3.69 ± 1.21	3.18 ± 1.19	> 0.05
SDMT score	53.0 ± 11.1	42.9 ± 9.9	** < 0.001**
BVMT-R score	27.0 ± 5.0	24.2 ± 5.5	** < 0.05**
CVLT-II score	53.3 ± 9.3	50.4 ± 9.3	> 0.05

Significant values are in [bold].

EDSS, Expanded Disability Status Scale; SDMT, Symbol Digit Modalities Test; BVMT-R, Brief Visuospatial Memory Test–Revised; CVLT-II, California Verbal Learning Test Second Edition; MS, multiple sclerosis.

*Student t-test for paired samples.

### Factors predicting medication adherence in patients with MS

Patient characteristics (sex, age, and education level), disease characteristics (disease duration and treatment duration), form of drug administration (injectable or oral), and scores of the Symbol Digit Modalities Test (SDMT) or Brief Visuospatial Memory Test–Revised (BVMT-R), or California Verbal Learning Test, Second Edition (CVLT-II) were included in the binary logistic regression analysis as independent variables. Dependent binary variables in the models were adherence (PDC ≥ 0.8) or nonadherence (PDC < 0.8). Table 4 shows the results of the binary logistic regression analysis, which identified significant factors that predict nonadherence to DMT in patients with MS.

**Table 4:** Results of binary logistic regression analysis for factors for predicting medication adherence.

Variable	Adherent/non-adherent				
	β	SE	Odds ratio	95% CI	p-value
SDMT score	0.097	0.031	1.10	1.04, 1.17	< 0.05
Self-injected drug	1.534	0.646	4.64	1.31, 16.46	< 0.05
Duration of treatment	1.624	0.751	5.07	1.16, 22.10	< 0.05
EDSS score	0.816	0.347	2.26	1.14, 4.46	< 0.05

SE, standard error; SDMT, Symbol Digit Modalities Test; EDSS, Expanded Disability Status Scale; CI, confidence interval.

Patients with a higher SDMT score, self-injection therapy, shorter treatment duration, and higher disability were more likely to be adherent to DMT.

## Discussion

In this study, overall adherence to DMT was low, with approximately 48% of patients not meeting the adherence criteria (PDC ≥ 0.8). The rate of adherence (52%) at 12 months was lower than that (60–77%) reported by other authors^17–19^, who applied the PDC criteria to larger samples. The disparate findings may have been due to differences between study populations (in other studies, investigators included patients with disability claims or patients before and after the first DMT claim date) or the DMTs analyzed^17,18^. Adherence rates vary among studies according to study sample and methods^11,13,17,18^, and it is apparent that adherence remains suboptimal in patients with MS initiating DMTs, and measures to improve adherence are warranted.

This study found several associations between patient characteristics and DMT adherence. Compliance and adherence levels to DMT were lower in men with MS than in women with MS. Other studies have provided mixed evidence regarding the difference in adherence between sexes^5,19,20^. Although MS is more prevalent in women than in men, it is important to focus on patient-centered care that can be used by health care practitioners to aid in enhancing adherence to DMT in men.

In the present study, oral DMT administration, a lower EDSS score, and longer treatment duration were associated with a greater lack of adherence. Many studies have compared adherence by type of DMT^5,19,21,22^. There is no consensus on which DMT patients have a higher compliance with: some studies have shown that patients using self-injected therapy, predominantly IFNβ, are more adherent than those not using such therapy^5,19^, other studies have indicated that patients using oral therapy, predominantly fingolimod, are more adherent than those not using such therapy^21,22^. Likewise, a study assessed three oral and five self-injected DMTs and found that the route of administration was not a significant predictor of nonadherence^23^. Given the equivocal evidence of the studies^5,19,21–23^, the difference in adherence between injectable and oral DMT remains unclear. In many studies, treatment adherence was found to be related to the duration of the treatment and neurological disability^24,25^. Similarly, in our study, patients with a longer treatment duration and lower EDSS score were also non-adherent to DMT.

Patients adherent to DMT (PDC > 80) in our study had a significantly decreased likelihood of relapse. The observed association between nonadherence and a higher probability of severe relapse (p < 0.05) coincides with the evidence demonstrated in other studies that nonadherence is a significant predictor of relapse^7,18,26–28^. Therefore, clues that promote adherence may improve the overall outcomes for patients with MS receiving DMT by reducing the frequency of relapses and disease progression.

Cognitive impairment in patients with MS as an important indicator of safe medication use should be assessed in patients with MS. The Brief International Cognitive Assessment for Multiple Sclerosis (BICAMS) was selected for cognition assessment in our study, as the BICAMS was recognized as a short, highly sensitive, and easily administered battery for patients with MS^29,30^. We found an association between a lower score of information processing speed and PDC < 80. There are no published data about adherence and the SDMT score, so this study is the first to examine the relationship between information processing speed and DMT adherence. The SDMT assessment, which is a quick and effective assessment of cognition^29,30^ can be performed before DMT initiation and can help improve adherence to DMT. The patients with impaired information processing speed on the oral or injectable DMT should be closer monitored during routine visits. Some studies have shown that patient support programs have a positive impact on adherence to DMT independent of the treatment duration on DMT^31,32^. It is important that the majority of patients also believe in this positive effect^31^. E-pills or e-injection medication devices (e.g., timers or alarm watches) also can help improve medication compliance in these patients^32^. After all efforts are taken, if the patient still remains non-adherent, other treatment options should be considered.

The present study has several limitations. First, cognition was only tested with the BICAMS, so other cognitive domains were not assessed. However, most cognitive tests, despite their sensitivity to MS, are time consuming and not routinely used in clinical settings. The aim of this study was to estimate and assess the impact of a cognitive tool that is readily available in most countries. Second, fatigue and depression, which are common comorbid conditions that have a great impact on cognition, were not assessed in the study. However, patients with severe fatigue and depression were excluded from the study.

## Conclusions

Patients with a higher SDMT score or who were receiving self-injection therapy, or had a shorter treatment duration, or higher disability were more likely to be adherent to DMT. Improving patients’ adherence level requires not only decision-making between patients and physicians and addressing side-effect profiles of medications, but it also requires cognition assessment before DMT administration. To minimize nonadherence in patients with MS, the patient’s information processing speed should be considered before DMT initiation, and appropriate treatment options should be discussed.

## Methods

### Study design and population

This prospective, cross-sectional study was performed at the Multiple Sclerosis Center of Vilnius University Hospital Santaros Klinikos, Lithuania. Patients were enrolled and assessed between 2018 and 2021.

A total of 98 patients were enrolled in this study. All patients had relapsing MS and were on DMT (injectable or oral therapy).

**Inclusion criteria** for all patients were as follows:

- Male or female patients older than 18 years of age;
- Patients diagnosed with MS according to the McDonald criteria^33,34^;
- Patients with a relapsing disease course;
- Patients receiving the same DMT at least 6 months before enrollment;
- Patients who had not used any cognition-influencing medication (e.g., antidepressants, neuroleptics, and anticholinergic drugs) at least 3 months prior to enrollment and during the study;
- Patients with no MS relapse or relapse treatment at least 3 months before enrollment and cognitive assessment; and
- Patients with MS who were fluent Lithuanian speakers.

**Exclusion criteria** for all patients were as follows:

- Patients with any neurologic or psychiatric disorders that could affect cognitive functions;
- Patients with a history of clinically significant central nervous system disease (e.g., stroke, traumatic brain, or spinal injury) or neurological disorders that could mimic MS;
- Patients with moderate or severe fatigue, anxiety, and/or depression; and
- Patients with neurological signs that could interfere with cognitive performance (e.g., optic neuritis, upper dominant extremity weakness, or severe ataxia).

### Neurological and cognitive assessment

The neurological assessment was performed in all participants, and neurological disability was assessed using the EDSS. The BICAMS was used for cognitive assessment^29,30^, which was performed by the same person in the same sequence:

- SDMT;
- BVMT-R, first three recall trials; and
- CVLT-II, first five trials. The Lithuanian version of the CVLT-II was used for assessment^35,36^.

### DMT and adherence

Eight different DMTs were identified and categorized into two groups: self-injected and oral. Self-injected therapies included IFNβ (Betaferon, Rebif, Avonex and Plegridy) and glatiramer acetate (Copaxone). Oral therapies included fingolimod, teriflunomide, and dimethyl fumarate.

Adherence was measured using pills or injections counts, which were combined into PDC. PDC was calculated for all patients as the sum of days during the follow-up period that were covered by pills or injections, divided by the number of days in the follow-up period (365 days)^37^. Values for PDC ranged from 0 to 100% with higher values indicating higher adherence and “100%” indicating a patient who had complete DMT adherence. The percentages of patients with adherence levels of < 80% were considered as non-adherent and > 80% as adherent.

### Statistical analysis

Descriptive statistics are presented as mean (m) and standard deviation. The Student t-test was used to compare means of the same variables between the two groups when the data distribution was normal. Categorical variables are expressed as absolute number and percentage. Categorical variables were analyzed using the chi-square test. To assess the normality of the distribution of quantitative variables, the Shapiro–Wilk test was used. In the regression analyses, adherence was modeled as a binary variable, with PDC ≥ 0.8 representing adherence and PDC < 0.8 indicating nonadherence. Explanatory variables (covariates) included age, sex, education level, disease duration, treatment duration, self-injectable or oral therapy, disability, SDMT score, or BVMT-R score, or CVLT-II score.

Data were analyzed using the statistical software package SPSS (version 23.0 for Windows, IBM Corp.) The level of statistical significance was set at p < 0.05.

### Ethics statements

The Lithuanian Bioethics Committee approved the study (date: January 27, 2011; number [no.]: L-12–01/2), and the Lithuanian Bioethics Committee granted permission to continue the study (date: February 22, 2018; no.: 6B-18–41). All methods were performed in accordance with the relevant guidelines and regulations. Written informed consent was obtained from all the participants prior to study inclusion.
